# Omi/HtrA2 Protease Associated Cell Apoptosis Participates in Blood-Brain Barrier Dysfunction

**DOI:** 10.3389/fnmol.2019.00048

**Published:** 2019-02-22

**Authors:** Yueyu Hu, Yong Bi, Danhua Yao, Pengfei Wang, Yousheng Li

**Affiliations:** ^1^Department of Neurology, Shanghai Fourth People’s Hospital affiliated to Tongji University School of Medicine, Shanghai, China; ^2^Department of General Surgery, Shanghai Ninth People’s Hospital, Shanghai Jiaotong University School of Medicine, Shanghai, China

**Keywords:** sepsis, blood-brain barrier, hCMEC/D3 cells, UCF-101, Omi/HtrA2

## Abstract

**Background**: Omi/HtrA2 is a proapoptotic mitochondrial serine protease involved in caspase-dependent cell apoptosis, translocating from mitochondria to the cytosol after an apoptotic insult. Our previous study indicated pre-treatment with UCF-101, a specific inhibitor of Omi/HtrA2, could significantly reduce neuronal apoptosis and attenuate sepsis-induced cognitive dysfunction. Various hypotheses involving blood-brain-barrier (BBB) disruption have been proposed to account for sepsis-associated encephalopathy (SAE). Here, we attempted to explore whether interference of Omi/HtrA2 by RNA interference or UCF-101 pre-treatment can improve sepsis-induced disruption of BBB using human cerebral microvascular endothelial cell line (hCMEC/D3) *in vitro* and if so, to explore mechanisms involved Omi/HtrA2 protease mediates BBB disruption in SAE.

**Methods**: hCMEC/D3 cell monolayers were intervened by different concentrations of LPS (0–50 μg/mL) over experimental period. Pharmacological or gene interventions (by silencing RNA of Omi/HtrA2) were used to study molecular mechanisms involved in sepsis-associated Omi/HtrA2 translocation, cell apoptosis and BBB dysfunction. BBB function was assessed by trans-endothelial electrical resistance (TEER) and permeability to labeled dextrans (FITC-4kDa). Tight junction (TJ) integrity was assessed by immunofluorescence, western blotting and transmission electron microscopic (TEM) analyses. Apoptosis was determined using flow cytometry and TUNEL assay. Mitochondrial membrane potential (MMP) and oxidative stress were also investigated.

**Results**: LPS affects hCMEC/D3 TJ permeability in a concentration- and time-dependent manner. LPS intervention resulted in a significant disruption of BBB, as manifested by decreased TEER (by ~26%) and a parallel increased paracellular permeability to FITC- (4kDa) dextrans through hCMEC/D3 monolayers. The inhibition of Omi/HtrA2 by UCF-101 or Omi/HtrA2 shRNA reduced LPS-induced brain endothelial cell apoptosis, and resulted in significant improvement on LPS-induced BBB disruption as well as decreased occludin, claudin-5 and ZO-1 expressions. Omi/HtrA2 manipulated endothelial cell apoptosis by shifting into cytosol and inducing X-linked inhibitor of apoptosis protein (XIAP) degradation. UCF-101 administration or Omi/HtrA2 shRNA intervention did attenuate the degradation of XIAP, Poly ADP-ribose polymerase (PARP) cleavage, and caspase-3 cleavage. However, only UCF-101 partly prevented the mobilization of Omi/HtrA2 from the mitochondria to the cytosol after LPS intervention. That abrogation of Omi/HtrA2 by UCF-101 or Omi/HtrA2 shRNA resulted in a significant improvement on LPS-induced decrease of MMP. Oxidative stress was significantly increased in the LPS treated group compared to the control or NC-shRNA group. However, abrogation of Omi/HtrA2 by UCF-101 or Omi/HtrA2 shRNA did not significantly improve oxidative injury.

**Conclusions**: Our study indicated an important role of Omi/HtrA2 in manipulating LPS-induced cell apoptosis and BBB integrity by translocating from mitochondria into cytosol in brain endothelial cells. Omi/HtrA2 induced mitochondrial pathway apoptosis, which involves inhibition of an important antiapoptotic protein XIAP and influence on MMP. Therapeutic methods that inhibit Omi/HtrA2 function may provide a novel therapeutic measure to septic encephalopathy.

## Background

Sepsis-associated encephalopathy (SAE), which results in cognitive impairment, is a common complication of systemic sepsis (Varatharaj and Galea, 2017). Various hypotheses involving blood-brain-barrier (BBB) dysfunction have been proposed to account for SAE (Osburg et al., 2002; Banks, 2005; Sharshar et al., 2007). Recently, many studies had demonstrated that endothelial cell apoptosis induced by systemic inflammation may bring about barrier disruption and eventually lead to septic encephalopathy (de Vries et al., 1996; Cardoso et al., 2012; Chaudhry and Duggal, 2014; Wang et al., 2015). Features include cell membrane abnormalities, mitochondrial damage and endothelial apoptosis (Karahashi et al., 2009).

The process of apoptosis or programmed cell death is manipulated by aspartate-specific cysteine proteases known as caspases, which is regulated by the death receptor pathway or the mitochondrial pathway (Kumar and Vaux, 2002). The Inhibitors of apoptosis proteins (IAPs) serve as endogenous inhibitors of cell apoptosis *via* inhibiting both caspase-9 and caspase-3 activation (Vaux and Silke, 2003). Studies have demonstrated that besides cytochrome c and procaspases, mitochondria contain several other proapoptotic molecules that are released during apoptosis, including the Smac/DIABLO and the mitochondrial serine protease Omi/HtrA2, which bind to X-linked inhibitor of apoptosis protein (XIAP) and result in their displacement from activated caspases, thus promoting caspase-dependent apoptosis (van Loo et al., 2002; Vaux and Silke, 2003).

Omi/HtrA2 is formed as a precursor that translocates to the mitochondria, and after an apoptotic insult is released to the cytosol. Unlike Smac/DIABLO, whose pro-apoptotic effect involved its physical binding with IAPs, Omi/HtrA2 induced apoptosis by its inhibition of IAPs protease activity and its direct binding with IAPs (Srinivasula et al., 2003; Yang et al., 2003). Omi/HtrA2’s relative effect of IAP binding compared with serine protease activity of remains unclear, which probably depends on cell and stimulation types. The protease activity of Omi/HtrA2 can be depressed by a selective inhibitor, UCF-101 (Cilenti et al., 2003). It has been suggested that UCF-101 decreases apoptosis in many vitro and *in vivo* studies (e.g., S-nitrosoglutathione induced apoptosis in human endothelial cells (Liu et al., 2010). It had been also demonstrated that Omi/HtrA2-knockdown can protect cell from all kinds of apoptotic stimuli (Hegde et al., 2002; Martins et al., 2002). Furthermore, some studies have proved that higher level of Omi/HtrA2 distinctly promoted apoptosis (Martins et al., 2002; Cilenti et al., 2003). Our previous study indicated pre-treatment with UCF-101 could significantly reduce neuronal cell apoptosis and attenuate sepsis induced cognitive dysfunction (Hu et al., 2013). But, the molecular mechanism has not been studied and it has also not demonstrated whether suppression of Omi/HtrA2 expression level can improve BBB disruption induced by sepsis.

It has been demonstrated that UCF-101 can reduce apoptosis and protect organ functions in some kinds of pathologic condition including cerebral ischemia/reperfusion injury, cardiomyocyte dysfunction, tubular fibrosis (Liu et al., 2005; Althaus et al., 2007; Kim et al., 2010).

The present study was aimed (Varatharaj and Galea, 2017) to manifest whether inhibition of Omi/HtrA2 by RNA interference or UCF-101 treatment could improve BBB disruption induced by sepsis *in vitro*; (Banks, 2005) if so, to probe the mechanisms by which Omi/HtrA2 induces BBB dysfunction and septic encephalopathy.

## Materials and Methods

### Antibodies and Chemicals

Antibodies used in this study were pursued from the following companies: rabbit anti-cleaved-caspase-3, anti-Poly ADP-ribose polymerase (anti-PARP), anti-XIAP and anti-HtrA2 from Santa Cruz Biotechnology (Santa Cruz, CA, USA); Rabbit anti-ZO-1, anti-claudin 5 and anti-occludin from Abcam (Cambridge MA, USA); Goat anti-rabbit conjugated to Alexa Fluor 488, MitoTracker^®^ Green FM and Tetramethylrhodamine, methyl ester (TMRM) from Thermo Fisher Scientific, Waltham, MA, USA; Rabbit anti-Cox IV and other secondary antibodies were purchased from Cell Signaling Technology (Danvers, MA, USA). Fluorescein isothiocyanate (FITC)- dextrans were purchased from Sigma-Aldrich. UCF-101 was purchased from Calbiochem (Darmstadt, Germany). Sterile culture ware was purchased from GibcoInc (Grand Island, NY, USA) while other molecular biology grade chemicals were obtained from Sigma-Aldrich (St. Louis, MO, USA) or Bio-Rad laboratories (Hercules, CA, USA).

### Cell Culture

The cell line of human cerebral microvascular endothelial cell (HCMEC/D3) has been widely used *in vitro* for studying molecular mechanism of BBB (Sajja et al., 2014, 2015). The cell line was purchased from Cedarlane Laboratories (Hornby, ON, Canada) and cells (passages 28–31) were seeded on collagen coated small well chamber slides, 6-well plates or 75 cm^2^ flasks with a density of  2 × 10^4^/cm^2^ and cultured at the whole EBM-2 medium at 37°C (Sajja et al., 2015). The culture medium was replaced every 3 days until the monolayer became confluent. The verification of cell monolayer and tight junction (TJ) formation were evaluated by measuring trans-endothelial electrical resistance (TEER), which ranged from 80 to 120 Ω cm^2^.

### Construction of Omi/HtrA2 shRNA Lentivirus Vector

Omi/HtrA2 shRNA lentivirus vector was constructed using the shRNA design program according to the principle of RNA interference design sequence. The shRNA target sequences were Sense: 5′-GGGGAGUUUGUUGUUGCCAdTdT-3′; Anti-sense: 5′-UGGCAACAACAAACUCCCCdTdT-3′. The double-stranded DNA were subsequently composed by the above sequences. Then the pLKO.1-EGFP vector (Western Biotechnology, Chongqing, China) was sliced into linear with Age I and EcoR I enzyme (Fermentas, Thermo Scientific, Waltham, MA, USA) and the double-stranded DNA was connected with vector using 1% agarose gel electrophoresis, which was transformed into competent DH5α cells. The pLKO.1-EGFP plasmid can express green fluorescent protein (GFP) and after the positive clones-sequence was verified, the lentiviral plasmids were generated by recombining the pCMV-VSV-G, pCMV-dR8.2 and pLKO.1-EGFP plasmids. The shRNA was then transfected into 293T cells (Shanghai Research Institute, Chinese Academy of Sciences, Beijing, China) according to the instruction for Lipofectamine^®^ 2000 (Invitrogen, Carlsbad, CA, USA). Then, the cell supernatants and extracts were collected after incubation with transfection complexes for another 48 h. The transfection results were observed using a fluorescence microscope (Leica Microsystems, Wetzlar, Germany).

### Construction of Stably Silencing Omi/HtrA2 Gene hCMEC/D3 Cell Line

We then concentrated the virus and infected it into HCMEC/D3 cells. The expression of GFP was sorted by flow cytometry (Beckman, Brea, CA, USA) after cell culture. The positive expression showed the success of the infection process. The fluorescence quantitative polymerase chain reaction (qPCR; Eppendorf, Hamburg, Germany) and Western blot confirmed that the Omi/HtrA2 mRNA and protein expression in infected HCMEC/D3 cells decreased significantly. Specific methods for Western blot and Real time PCR (RT-PCR) were mentioned later. The result confirmed that HCMEC/D3 cell line expressing stably silenced Omi/HtrA2 was established.

### Assessment of BBB Integrity

HCMEC/D3 cells were cultured with the transwell chamber as mentioned previous. Cells were seeded in the apical chambers of 24 or 12-well Transwell inserts with a volume of 150 or 400 μL, respectively. Transwell inserts were then placed in suitable culture plates. Cell monolayer integrity was assessed by TEER, paracellular permeability to FITC-4kDa and transmission electron microscopic (TEM) analyses. TEER has been extensively used to assess TJ function of the endothelial cells in BBB models (Hatherell et al., 2011). TEER was measured when the confluent monolayer was formed using Millicell-ERS equipment. TEER values were calculated as Ωcm^2^ by multiplying the surface area of the Transwell insert. Dextran assay for measurement of cell permeability was applied (Sajja et al., 2015). For TEM analysis, cells were fixed with 2.5% glutaraldehyde for 2 h at 4°C and subsequently with 1% OsO4–0.15M Na cacodylate/HCL (pH7.4) for 30 min. The cells were then dehydrated in graded ethanol and polymerized at 60°C for 48 h.

### Treatment

Briefly, cells were exposed to different concentrations of LPS (0–50 μg/mL) over a 24-h experimental period. In separate experiments, hCMEC/D3 cells were pretreated with 25 or 50 μM UCF-101 (Klupsch and Downward, 2006), for 1 h prior to and for the duration of LPS exposure. Drugs were dissolved in DMSO at final concentration <0.1%.

### Immunofluorescence

TJ protein expression was analyzed by immunofluorescence procedure. Briefly, hCMEC/D3 cells cultured on plates were fixed, permeabilized and subsequently blocked with 5% goat serum at room temperature for 30 min; and the cells were incubated with primary antibodies (1:100–150) overnight at 4°C. Then, cells were incubated for 1 h at room temperature with Alexa Fluor 594-conjugated secondary antibodies, respectively. Cells were rinsed with PBS and then mounted with DAPI. Images were captured in inverted fluorescence microscope.

### Real Time PCR (RT-PCR)

RT-PCR was performed as described previous with slight modification (Qin et al., 2015). Briefly, total RNA was extracted from hCMEC/D3 cells using TRIzol reagent (Invitrogen, Carlsbad, CA, USA) according to protocol of the manufacturer. Complimentary DNA (cDNA) was synthesized from 100 to 500 ng of total RNA using the ReverTra Ace qPCR RT Kit (TOYOBO, Osaka, Japan). Gene expressions in the samples were assayed based on SYBR green florescence method. Thermocycling was performed as follows: 95°C for 10 min; 40 cycles of 95°C for 10 s and 60°C for 30 s; and 72°C for 10 s. The 5′ forward and 3′ reverse complement PCR primers for amplification of HtrA2 were, CTAGCTAGCGCCACCATGGCTGCGCCGAGGGC and CGACGCGTTCATTCTGTGACCTCAGGGGTCACA, respectively. The target gene expression (Ct value) of each sample was normalized with glyceraldehyde-3-phosphate dehydrogenase. All reactions were repeated three times and the data were analyzed by the 2^−ΔΔCt^ method (Livak and Schmittgen, 2001).

### Western Blotting

Detailed procedure followed the previous description (Sajja et al., 2015). Briefly, hCMEC/D3 cells were harvested and total proteins were extracted using RIPA Extraction Reagents (ProMab Biotechnology, Los Angeles, CA, USA). Then, protein content in cell lysates was determined by bicinchoninic acid assay and equal amount of denatured protein was subjected to SDS-PAGE (5–12% acrylamide denaturing gel). Bands were electrotransferred to PVDF membranes for 90 min at 220 mA (or 30 V, overnight transfer), blocked and incubated with primary antibodies overnight (dilution range: 1:1,000–1:500) in blocking buffer. Blots were washed and incubated with HRP-conjugated secondary antibody (1:10,000) for 2 h at RT. The immunoreactive proteins on the membrane were visualized with an enhanced chemiluminescent detection system (Pierce, Rockford, IL, USA). Mitochondrial and cytosolic fractions were separated as described previously (Kim et al., 2009). Briefly, samples were homogenized and centrifuged. Then the supernatant was centrifuged at 15,000 *g* for 30 min and the cytosolic fraction was collected and stored at −70°C. The pellet was washed twice with sucrose buffer and was suspended in PBS containing 0.1% Triton X-100. Then the pellet was disrupted twice with a sonicator and centrifuged at 15,000 *g* for 30 min. The mitochondrial fraction was collected and stored at −70°C. Cox IV was used as protein loading for the mitochondrial proteins preparations (Sajja et al., 2015).

### Flow Cytometric Detection

The percentages of apoptosis and necrosis cells were estimated using Dead Cell Apoptosis Kit with Annexin V Alexa Fluor^®^ 488 for flow cytometry according to the manufacturer’s instructions (Invitrogen, Carlsbad, CA, USA). Cells were washed twice with cold PBS after been harvested, and then were incubated with FITC-Annexin V and PI working solution for 15 min. Cellular fluorescence was measured by flow cytometry analysis (FACS CaliburTM, BD Biosciences, San Jose, CA, USA).

### TUNEL Assay

TUNEL assay was performed by *in situ* cell death detection kit-POD (TUNEL). After treatment, hCMEC/D3 cells were washed and fixed with 4% paraformaldehyde in PBS for 20 min at 37°C and permeabilized with 0.1% Triton X-100 in PBS for 5 min. The following procedure for staining using the TUNEL reaction mix was performed following the manufacturer’s protocol (Roche Applied Science, Mannheim, Germany).

### Mitochondrial Membrane Potential

The mitochondrial membrane potential (MMP) of cells was assessed by the lipophilic probe JC-1 (Sharath Babu et al., 2017; Jia et al., 2019). For fluorescence ratio detection, cells were collected after treatment, and resuspended in 0.5 ml cell culture medium. A total of  0.5 ml JC-1 staining working solution was added, followed by incubation in a dark place at 37°C for 30 min and centrifugation at 500× *g* and 4°C. Then the supernatant was discarded, and cells were washed followed by centrifugation and precipitation, and the supernatant was discarded. The fluorescence intensity of cells was detected using the flow cytometer. The results are expressed as the ratio of fluorescent intensity of  J-monomers to aggregates.

For fluorescence imaging, cells were labeled with 2.5 mM JC-1 for 15 min at 37°C after treatment. Following labeling, the cover slips were washed with PBS and covered onto glass microscopic slides and examined immediately. Images were captured in verted fluorescence microscope.

### Oxidative Stress Measurement

The protein concentration was determined using the BCA method. Oxidative stress was evaluated by measuring the production of malondialdehyde (MDA) with a lipid peroxidation assay kit (Beyotime, Shanghai, China). Catalase (CAT) activity, Myeloperoxidase (MPO) activity and Glutathione (GSH) assay was performed as previously described (Hu et al., 2013; Aborehab et al., 2017; Ellis-Hutchings et al., 2018). MPO activity in supernatant was measured and calculated from the absorbance (at 460 nm) changes resulting from decomposition of H_2_O_2_ in the presence of  o-dianisidine (Aborehab et al., 2017).

### Statistical Analyses

Data are reported as the mean±1.0 SD when appropriate. Repeated measurement analysis of variance was used to study the within group change overtime for TEER. Differences between groups were evaluated by one-way analyses of variance (ANOVA) test followed by Bonferroni test for other comparisons between groups or the pairwise comparison of every combination of group pairs. All analyses were conducted in SPSS13.0 (SPSS Inc., Chicago, IL, USA). Statistical significance was accepted as *P* < 0.05.

## Results

### LPS Induces hCMEC/D3 Cell Apoptosis and Affects BBB Permeability in a Time- and Dose-Dependent Manner

**Figure 1:**
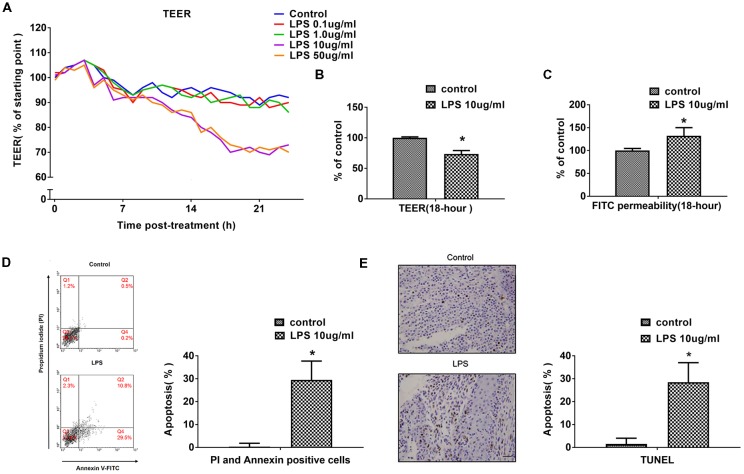
LPS induces human cerebral microvascular endothelial cell line (hCMEC/D3) cell apoptosis and affects blood-brain-barrier (BBB) permeability in a dose- and time-dependent manner. **(A)** The effects of different concentrations of LPS (0–50 μg/mL) on BBB integrity was determined *in vitro* by measuring trans-endothelial electrical resistance (TEER) over a 24-h experimental period. **(B)** Effects of 10 μg/mL LPS on TEER after 18-h treatment. **(C)** Effects of 10 μg/mL LPS on paracellular permeability to FITC- (4kDa) dextrans across hCMEC/D3 monolayers after 18-h treatment. **(D)** Effects of LPS on early cell apoptosis as determined by flow cytometry; quantitative analysis of the percentage of apoptotic cells was calculated. **(E)** Effects of LPS on cell apoptosis as determined by TUNEL assay. Apoptotic cells appear brown and the nucleus appear blue. Scale bar, 200 μm; quantitative analysis of the percentage of apoptotic cells was calculated. *P < 0.01, vs. control.

The effects of different concentrations of LPS (0–50 μg/mL) on BBB integrity were determined *in vitro* by measuring TEER and paracellular permeability to FITC- (4kDa) dextrans across hCMEC/D3 monolayers, over a 24-h experimental period. LPS (0, 0.1, 1, 10 or 50 μg/mL) induced a concentration-dependent and time-dependent drop in hCMEC/D3 TEER (Figure 1A). LPS caused a significant drop in TEER at a concentration of 10 μg/mL over time. Increasing the LPS concentration above 10 μg/mL caused however a similar drop in hCMEC/D3 TEER. In subsequent experiments, a LPS concentration of 10 μg/mL was used. Meanwhile, LPS did not have significant effect on hCMEC/D3 TEER up to 6 h, but caused a drop in TEER between 12 h and 24 h. The drop in TEER reached an obvious level by 18 h and increasing LPS-intervention time reached only a minimal additional drop at 24 h. So, LPS intervention time of 18 h was used in subsequent experiments, unless stated otherwise. We observed that LPS intervention resulted in a significant disruption of BBB, as manifested by reduced TEER (by ~26%) and a simultaneous increase in paracellular permeability of FITC- (4kDa) dextrans across hCMEC/D3 monolayers, as demonstrated in Figures 1B,C.

Previous study indicated that treatment with high pharmacological concentrations (50–100 μg/mL) of LPS not only decreased mRNA and protein expression levels of occludin and ZO-1, but also had effects on cell vitality (Qin et al., 2015). In present study, the impact of LPS on hCMEC/D3 cell apoptosis and necrosis were determined using flow cytometry and TUNEL assay. The proportion of apoptotic hCMEC/D3 cells was enhanced significantly after LPS intervention in comparison with the control group (Figures 1D,E).

### Omi/HtrA2 Participates in LPS-Induced Brain Endothelial Cell Apoptosis

**Figure 2:**
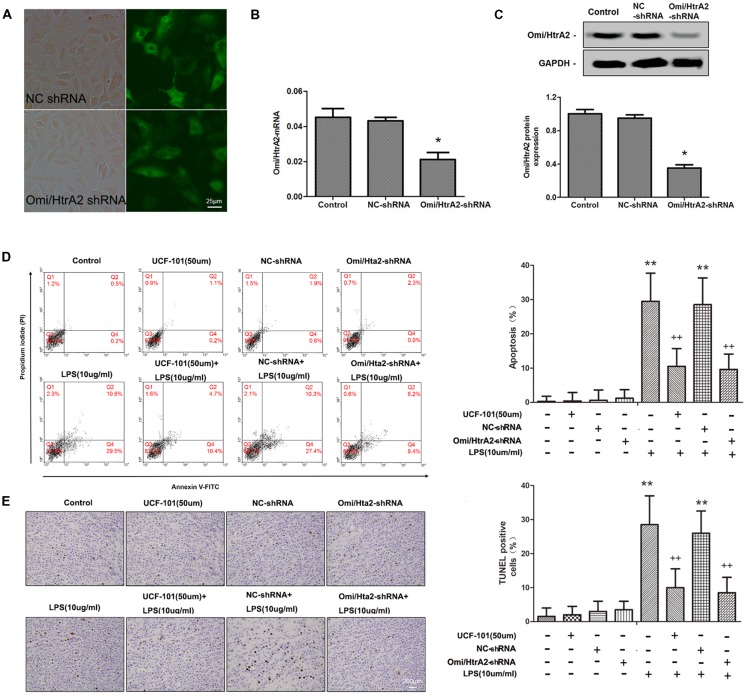
Omi/HtrA2 regulates LPS-induced brain endothelial cell apoptosis in LPS-treated hCMEC/D3 monolayers. **(A)** Fluorescent images of silencing Omi/HtrA2 gene HCMEC/D3 cell. Scale bar, 25 μm. **(B)** Omi/HtrA2 knock-down efficiency as determined by Rt-PCR or **(C)** western blot analyses in hCMEC/D3 cells transfected with Omi/HtrA2 shRNA lentivirus. **(D)** Effects of inhibition of Omi/HtrA2 by UCF-101 or Omi/HtrA2 shRNA on cell apoptosis as determined by flow cytometry; quantitative analysis of the percentage of apoptotic cells. **(E)** Effects of inhibition of Omi/HtrA2 by UCF-101 or Omi/HtrA2 shRNA on cell apoptosis as determined by TUNEL assay. Apoptotic cells appear brown and the nucleus appear blue. Scale bar, 200 μm; The percentage of apoptotic cells was calculated. *P < 0.05, vs. control or NC-shRNA, **P < 0.01, vs. control or NC-shRNA, ^++^*P* < 0.01, vs. control + LPS or NC-shRNA + LPS.

To verify the effect of Omi/HtrA2 on brain endothelial cell apoptosis, we specially knocked down Omi/HtrA2 expression by shRNA-based gene silencing technology or blocked the protease activity of Omi/HtrA2 by an inhibitor UCF-101. The Omi/HtrA2 knocked down efficacy in hCMEC/D3 cells after transfection were confirmed by the fluorescent images (Figure 2A), qRT-PCR (with ~53% decrease of Omi/HtrA2 expression; Figure 2B) and western blot (with ~65% decrease of Omi/HtrA2 expression; Figure 2C). The results of Annexin/PI analysis showed apoptotic hCMEC/D3 cells was increased significantly after intervention with LPS in comparison with the control group, which were dramatically reversed by UCF-101 or Omi/HtrA2 shRNA. There is no significant difference between UCF-101 plus LPS and Omi/HtrA2-shRNA+LPS groups as for apoptosis rate (Figure 2D). The data of Annexin/PI analysis was further confirmed by TUNEL assay (Figure 2E).

### Omi/HtrA2 Regulates LPS-Induced Loss of Brain Endothelial Barrier Integrity and Tight Junction Protein Expression

**Figure 3:**
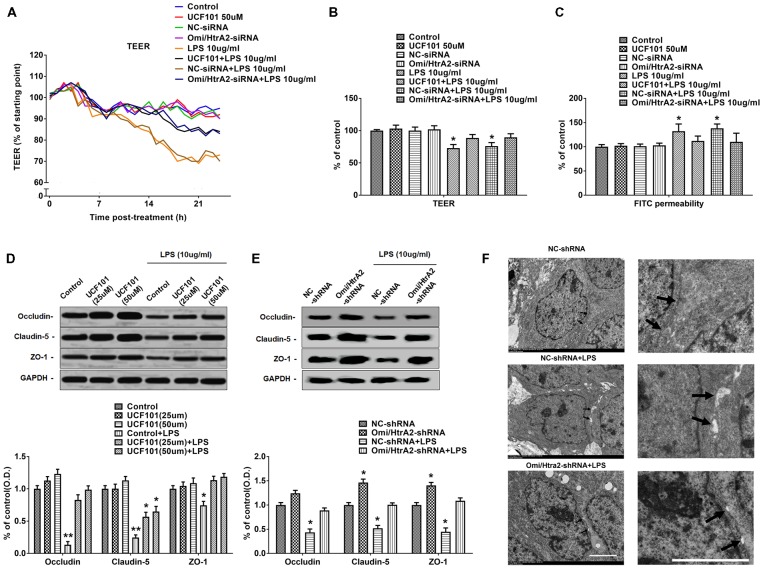
Omi/HtrA2 regulates LPS-induced loss of brain endothelial barrier integrity and tight junction (TJ) protein expression. **(A)** Effects of inhibition of Omi/HtrA2 by UCF-101 or Omi/HtrA2 shRNA on TEER in LPS-treated hCMEC/D3 monolayers over a 24-h experimental period. **(B)** Effects of inhibition of Omi/HtrA2 by UCF-101 or Omi/HtrA2 shRNA on TEER after 18-h treatment. **(C)** Effects of inhibition of Omi/HtrA2 by UCF-101 or Omi/HtrA2 shRNA on paracellular permeability to FITC- (4kDa) dextrans across hCMEC/D3 monolayers after 18-h treatment. **(D,E)** Effects of inhibition of Omi/HtrA2 by UCF-101 or Omi/HtrA2 shRNA on occludin, claudin-5 and ZO-1 expressions as determined by western blot analyses. Respective bands with GAPDH as loading control were shown. *P < 0.05, vs. control or NC-shRNA, **P < 0.01, vs. control or NC-shRNA. **(F)** Effects of abrogation of Omi/HtrA2 by Omi/HtrA2 shRNA on BBB ultrastructures in LPS-treated hCMEC/D3 monolayers as determined by transmission electron microscopic (TEM). Arrows show TJ. Scale bar, 10 μm.

**Figure 4:**
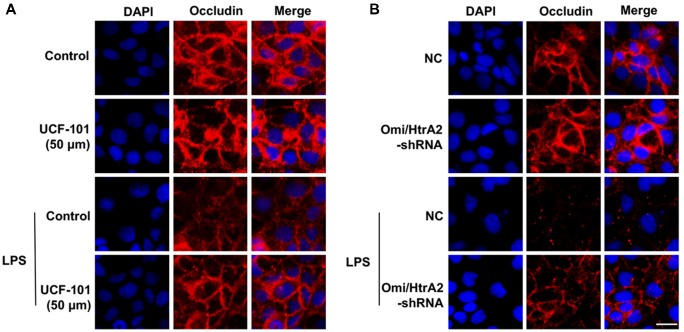
Effects of abrogation of Omi/HtrA2 by UCF-101 **(A)** or Omi/HtrA2 shRNA **(B)** on TJ protein occludin expression in LPS-treated hCMEC/D3 monolayers as determined by immunofluorescence. Red staining show TJ. Nucleus (blue) was labeled with DAPI. Scale bar, 25 μm.

**Figure 5:**
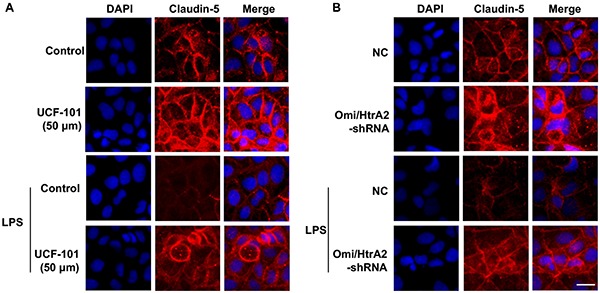
Effects of abrogation of Omi/HtrA2 by UCF-101 **(A)** or Omi/HtrA2 shRNA **(B)** on TJ protein claudin-5 expression in LPS-treated hCMEC/D3 monolayers as determined by immunofluorescence. Red staining show TJ. Nucleus (blue) was labeled with DAPI. Scale bar, 25 μm.

**Figure 6:**
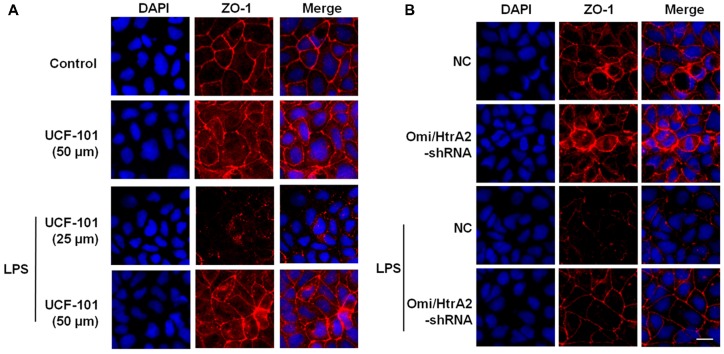
Effects of abrogation of Omi/HtrA2 by UCF-101 **(A)** or Omi/HtrA2 shRNA **(B)** on TJ protein ZO-1 expression in LPS-treated hCMEC/D3 monolayers as determined by immunofluorescence. Red staining show TJ. Nucleus (blue) was labeled with DAPI. Scale bar, 25 μm.

We further observed that abrogation of Omi/HtrA2 by UCF-101 or Omi/HtrA2 shRNA in brain microvascular endothelial cells resulted in a significant improvement on LPS-induced loss of BBB integrity, as demonstrated by similar TEER to control group and a parallel improvement in paracellular permeability to FITC- (4kDa) dextrans across hCMEC/D3 monolayers (Figures 3A–C). In line with the altered paracellular permeability, we demonstrate the effects of Omi/HtrA2 signal path on BBB endothelial TJ protein expression. As illustrated in Figures 3D,E, western blot analyses indicated that Omi/HtrA2 knock-down or inhibition abrogated LPS-induced decline of occludin, claudin-5 and ZO-1 expression level in hCMEC/D3 cells (vs. LPS). Cells were also processed for immunofluorescence localization of TJ protein including occludin, claudin-5 and ZO-1 (Figures 4–6). UCF-101 treatment alone did not alter TJ integrity or expression compared with the control group. However, an obvious increase in claudin-5 and ZO-1 as well as a non-significant increase in occludin expression is measured in the Omi/HtrA2 shRNA control group. LPS treatment induced occludin and claudin-5 junctional discontinuity and ZO-1 expression level decline in contrast with the control group. Treatment with UCF-101 or Omi/HtrA2 knock-down improved LPS-induced occludin and claudin-5 junction dis-integrity and ZO-1 expression inhibition. We further examined the BBB ultrastructures using TEM (3F). The TJs remained intact in NC-shRNA group and were unclear in NC-shRNA + LPS group. However, the TJ ultrastructure was significantly improved in Omi/HtrA2-shRNA + LPS group.

### Omi/HtrA2 Manipulates LPS-Induced Endothelial Cell Apoptosis Through Translocating From Mitochondria to Cytosol and Inducing XIAP Degradation

**Figure 7:**
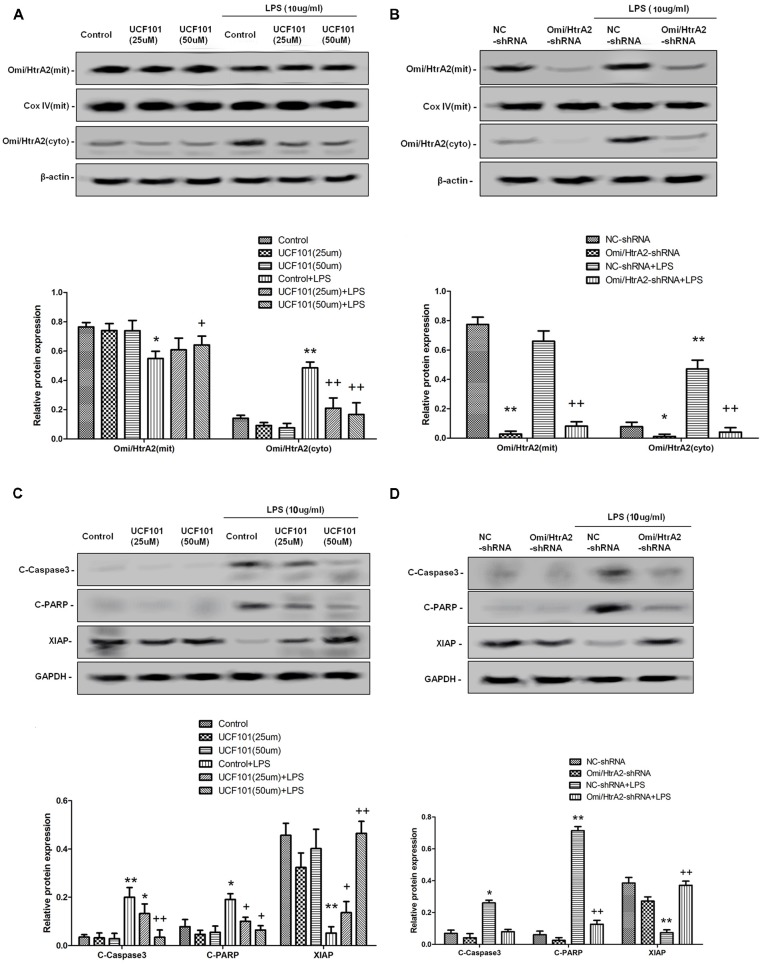
Omi/HtrA2 regulates LPS-induced endothelial cell apoptosis by translocating from mitochondria into cytosol *via* proteolytic degradation of proteins that can normally inhibit caspase activity. **(A)** Effects of Omi/HtrA2 protease activity inhibition by UCF-101 or **(B)** Omi/HtrA2 silencing by Omi/HtrA2 shRNA on Omi release into the cytoplasm in LPS-treated hCMEC/D3 monolayers. Respective bands with β-actin as loading control were shown. **(C)** Effects of Omi/HtrA2 inhibition by UCF-101 or **(D)** Omi/HtrA2 silencing by Omi/HtrA2 shRNA on degradation of X-linked inhibitor of apoptosis protein (XIAP), Poly ADP-ribose polymerase (PARP) cleavage, and caspase-3 cleavage in LPS-treated hCMEC/D3 monolayers. Respective bands with GAPDH as loading control were shown. *P < 0.05, vs. control or NC-shRNA, **P < 0.01, vs. control or NC-shRNA, ^+^*P* < 0.05, vs. control + LPS or NC-shRNA + LPS, ^++^*P* < 0.01, vs. control + LPS or NC-shRNA + LPS.

Studies have verified that Omi/HtrA2 promotes apoptosis through translocating from mitochondria into cytosol and inducing XIAP degradation. To assess the location of Omi/HtrA2, we separated mitochondrial and cytosolic fractions. As demonstrated in Figure 7, in the control group, Omi/HtrA2 protein exists almost exclusively in the mitochondria, and very little or none exists in the cytoplasm. After LPS treatment, Omi/HtrA2 (cyto) expression level in cytoplasm increased obviously, meanwhile Omi/HtrA2 (mito) expression level in mitochondria was decreased, which indicated that LPS induced mobilization of Omi/HtrA2 from the mitochondria into the cytosol.

Omi/HtrA2 promotes apoptosis through inhibition caspase activity. Unlike Smac/DIABLO, Omi/HtrA2 not only directly binds to XIAP *via* its reaper motif but also induces XIAP degradation by its protease activity (Srinivasula et al., 2003; Yang et al., 2003). To determine whether Omi/HtrA2 activation is important for the apoptosis that follows LPS, cells were treated with UCF-101, an inhibitor of the proteolytic activity of Omi/HtrA2. The administration of UCF-101 did attenuate the degradation of XIAP, PARP cleavage, and caspase-3 cleavage (Figure 7C). Meanwhile, UCF-101 also partly prevented the mobilization of Omi/HtrA2 from the mitochondria to the cytosol after LPS treatment (Figure 7A).

In line with UCF-101 administration regulating Omi/HtrA2 activation and its downstream targets, we also demonstrate the effects of Omi/HtrA2 shRNA intervention. As illustrated in Figure 7B, although Omi/HtrA2 shRNA intervention resulted in decreased Omi/HtrA2 expression in both mitochondria and cytosol, but did not prevent the mobilization of Omi/HtrA2 from the mitochondria to the cytosol after LPS treatment. However, Omi/HtrA2 shRNA intervention did attenuate the degradation of XIAP, PARP cleavage, and caspase-3 cleavage as well (Figure 7D).

### Omi/HtrA2 Influences Mitochondrial Membrane Potential Which Involved in LPS-Induced Endothelial Cell Apoptosis

**Figure 8:**
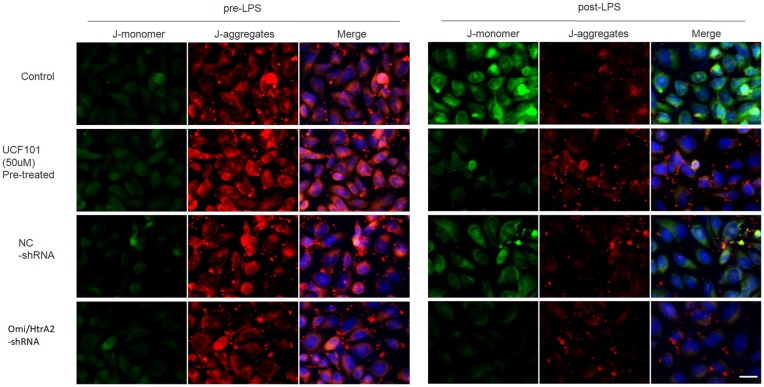
Effects of inhibition of Omi/HtrA2 by UCF-101 or Omi/HtrA2 shRNA on mitochondrial membrane potential (MMP) in LPS-treated hCMEC/D3 monolayers as determined by JC-1 staining using fluorescence microscope. Cells stained with red color indicates high MMP and green color indicates low MMP. Scale bar, 25 μm.

**Figure 9:**
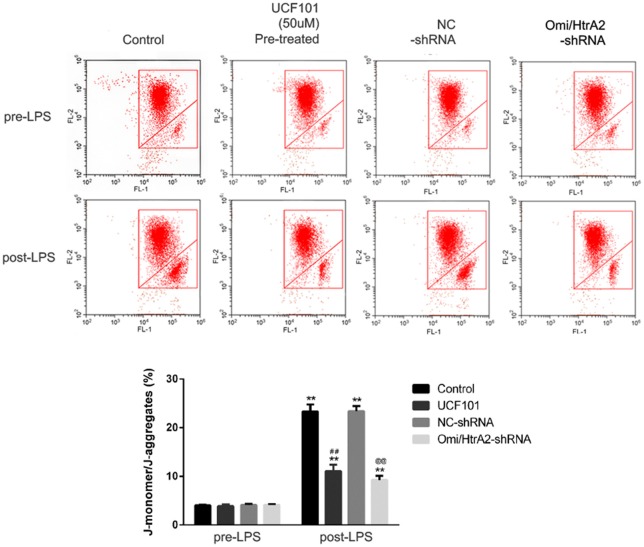
Effects of inhibition of Omi/HtrA2 by UCF-101 or Omi/HtrA2 shRNA on MMP in LPS-treated hCMEC/D3 monolayers as determined by JC-1 staining using flow cytometric detection. The results are expressed as the ratio of J-monomers to aggregates. **P < 0.01, vs. pre-LPS, ^##^*P* < 0.01, vs. control, ^@@^*P* < 0.01, vs. NC-shRNA.

Since apoptosis is accompanied by fission of mitochondria and loss of transmembrane potential may appear in the early apoptotic process (Youle and van der Bliek, 2012), and as previously shown we verified that UCF-101 could partly inhibit the translocation of Omi/HtrA2 from the mitochondria to the cytosol, and we next investigated whether Omi/HtrA2 intervention affect MMP utilizing JC-1 staining. hCMEC/D3 cells were incubated with JC-1 (Figure 8), which emerged either as a green-fluorescent at depolarized membrane potentials or as a red-fluorescent J-aggregate at hyperpolarized membrane potentials. When hCMEC/D3 cells was intervened with LPS, there was decrease in red fluorescence concentration and exhibited enhanced green fluorescence manifesting that all JC-1 remained in monomeric form. Intervening with UCF-101 or Omi/HtrA2 shRNA resulted in a significant improvement on LPS-induced loss of MMP. The ratio of fluorescent intensity of J-monomers to aggregates determined using flow cytometry was consistent with the above results (Figure 9).

### Oxidative Stress Did Not Contribute to Alleviating Apoptosis Through Omi/HtrA2 Intervention

**Figure 10:**
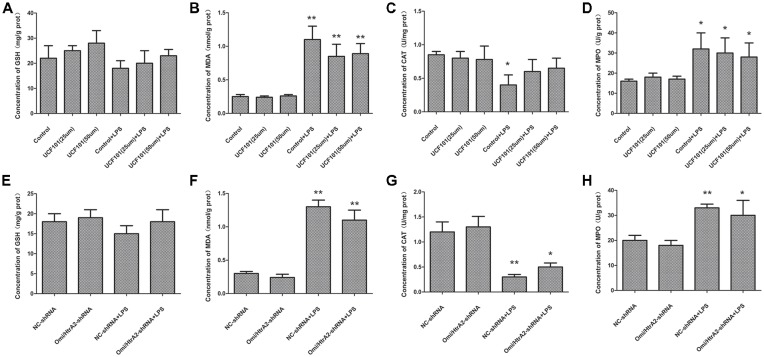
Oxidative stress did not play a key role in alleviating apoptosis by Omi/HtrA2 intervention. **(A–D)** Effects of Omi/HtrA2 inhibition by UCF-101 or **(E–H)** Omi/HtrA2 shRNA on GSH, malondialdehyde (MDA), catalase (CAT) and myeloperoxidase (MPO) levels in LPS-treated hCMEC/D3 monolayers. *P < 0.05, vs. control or NC-shRNA, **P < 0.01, vs. control or NC-shRNA.

Mitochondrial damage was always associated with oxidative stress, so we verified whether oxidative stress participated in LPS-induced endothelial cell apoptosis regulated by Omi/HtrA2. The oxidative stress damage parameters such as MDA, GSH, MPO and CAT levels are demonstrated in Figure 10. The density of MDA and MPO activity, were obviously increased in the LPS group compared to the control or NC-shRNA group. However, inhibition of Omi/HtrA2 through UCF-101 or Omi/HtrA2 shRNA did not significantly decrease MDA concentration or MPO activity. Meanwhile, LPS treatment significantly decreased CAT levels compared to the control or NC-shRNA animals, and Omi/HtrA2 inhibition by UCF-101 or Omi/HtrA2 shRNA slightly alleviate it. Compared with Control group, the GSH content of Control + LPS group somewhat decreased, but the difference was not significant.

## Discussion

We have demonstrated for the first time that Omi/HtrA2 regulates LPS-induced cell apoptosis by translocating from mitochondria into cytosol in brain endothelial cells. Using stably silencing Omi/HtrA2 gene HCMEC/D3 cell line or a selective Omi/HtrA2 protease inhibitor UCF-101, we have demonstrated that Omi/HtrA2 translocation induced to LPS-induced brain endothelial cell apoptosis and that knockdown of Omi/HtrA2 expression or inhibition of Omi/HtrA2’s protease activity decreased LPS-induced brain endothelial cell apoptosis, and further regulated LPS-induced brain blood barrier disruption. In addition, we have demonstrated that Omi/HtrA2 induced apoptosis mainly through a mitochondrial pathway, which include inhibition of an important antiapoptotic protein, XIAP and influence on MMP. Omi/HtrA2 might promote apoptotic cell death by both postmitochondrial and premitochondrial mechanism. Furthermore, oxidative stress did not play a critical effect in alleviating apoptosis induced by Omi/HtrA2 inhibition.

To verify whether Omi/HtrA2 may induce apoptosis in LPS treated hCMEC/D3 cells through inhibition of XIAP, we detected XIAP levels in LPS treated hCMEC/D3 cells. LPS can inhibition XIAP significantly, which was improved by UCF-101. Also, Omi/HtrA2 shRNA intervention did attenuate the degradation of XIAP, PARP cleavage, and caspase-3 cleavage as well. These results demonstrated that cytosolic Omi/HtrA2 induce apoptosis in sepsis through XIAP inhibition and subsequent caspase activation. In present study, we also found that UCF-101 could reduce Omi/HtrA2 translocation to the cytosol, besides inhibit Omi/HtrA2 enzyme activity in LPS treated endothelial cells. The critical role of UCF-101 on caspase cleavage and degradation of XIAP should be considered partly due to the inhibition of Omi/HtrA2 translocation.

Recent studies have proved that Omi/HtrA2 promote cell apoptotic by postmitochondrial mechanism involved inhibition of XIAP, but not premitochondrial pathway included increase of mitochondrial permeability (Cilenti et al., 2003; Liu et al., 2005; Kim et al., 2010). They proposed that Omi/HtrA2’s interaction with IAP which induced its catalytic degradation, causing irreversible inactivation of IAPs, but fails to increase cytochrome c release. Since opening of the mitochondrial permeability transition pore has been demonstrated to induce depolarization of the transmembrane potential, we investigated MMP utilizing JC-1 staining. Our present results demonstrated that abrogation of Omi/HtrA2 by UCF-101 or Omi/HtrA2 shRNA resulted in significant improvement on MMP, and thus, argued against Omi/HtrA2’s promoting apoptosis only at a post-mitochondrial level. The more immediate result in this study was that UCF-101 could partly prevent mobilization of Omi/HtrA2 from the mitochondria to the cytosol after LPS treatment. In line with our results, a previous *in vitro* study demonstrated that Omi/HtrA2’s translocation from mitochondria to the cytosol induced the outer mitochondrial membrane permeability and caspase activation in HeLa cells (Suzuki et al., 2004). these studies indicated that Omi/HtrA2 leads to caspase activation by some different pathways. A recent study reported *in vitro* mitochondrial Omi/HtrA2 higher expression level promoted cardiomyocyte apoptosis, not involved its translocation from the mitochondria, which might be depended by the inhibitions of the anti-apoptotic mitochondrial protein HAX-1 (Wang et al., 2016). So, the mechanisms involved in mitochondrial dysfunction induced by Omi/HtrA2 should be investigated further.

The Omi/HtrA2’s pro-apoptotic effect in varieties of pathology has been studied extensively, and the studies also suggest that the mechanism of Omi/HtrA2 pro-apoptotic effect is complicated. Sepsis-induced neuron death was also induced by the mitochondria-derived reactive oxygen species (ROS). It has been verified that the Omi/HtrA2 can affect the ROS system (Suzuki et al., 2004; Liu et al., 2010), for example inhibition of MnSOD, which should be involved in septic encephalopathy. Our previous study also indicated pre-treatment with UCF-101, could significantly reduce cerebral oxidative injury and attenuate sepsis induced cognitive dysfunction (Hu et al., 2013). In this study, we additionally observed whether oxidative stress promotes LPS-induced endothelial cell apoptosis and whether it was regulated by Omi/HtrA2. We confirmed that abrogation of Omi/HtrA2 by UCF-101 or Omi/HtrA2 shRNA in hCMEC/D3 monolayers could not significantly improve oxidative stress induced by sepsis *in vitro*. Therefore, oxidative stress might not play a critical effect in alleviating endothelial cell apoptosis during Omi/HtrA2 intervene.

Brain microvascular endothelial cell apoptosis was indeed associated with BBB dysfunction (Chen et al., 2017). However, there remains the confusion that whether the increased permeability of the cell monolayer after LPS exposure is simply a reflection of cell loss, rather than disruption of TJs. In immune-mediated colitis, apoptosis independent TJ permeability increases occur early and as disease progresses, apoptosis dependent barrier loss plays an important role (Su et al., 2013). However, it is unknown in brain barrier dysfunction. The concentration of LPS used in this study almost not affects cell vitality (Qin et al., 2015), indicated that apoptosis induced in this study might most be in early stage. We suppose that apoptosis independent and dependent barrier loss were both involved in the pathophysiology of this study. Anyhow, this suggests that Omi/HtrA2 protease associated cell apoptosis may participate in sepsis induced BBB dysfunction and septic encephalopathy. There are also some limitations. Cells were pre-treated with UCF-101 for 1 h prior to LPS incubation in this study. In many cases prevention is more effective than therapeutic strategy. In clinical, sepsis encephalopathy also basically relies on prevention. As a therapeutic strategy, the effect of UCF-101 on BBB integrity after LPS treatment could be investigated further.

In summary, our study indicated an important effect of Omi/HtrA2 in manipulating LPS-induced cell apoptosis and BBB integrity by translocating from mitochondria into cytosol in brain endothelial cells. Omi/HtrA2 induces apoptosis by a mitochondrial pathway cell apoptosis mechanism, which includes inhibition of a critical antiapoptotic protein, XIAP and influence on MMP. Therapeutic measures which include inhibition of Omi/HtrA2 expression or protease activity (for example gene therapy or inhibitor UCF-101) may provide an effective method to treat septic encephalopathy or decrease its susceptibility.

## Author Contributions

YB and YH carried out pharmacological or gene manipulation (by silencing RNA of Omi/HtrA2) approaches, BBB integrity assessment, western-blot and rt-pcr. YL and PW participated in the design of the study and performed the statistical analysis. YH and DY carried out flow cytometry and imunofluorescence and drafted the manuscript.

## Conflict of Interest Statement

The authors declare that the research was conducted in the absence of any commercial or financial relationships that could be construed as a potential conflict of interest.
